# The impact of opening the export promotion agencies on Indonesia's non-oil and gas exports

**DOI:** 10.1016/j.heliyon.2021.e07756

**Published:** 2021-08-13

**Authors:** Shochrul Rohmatul Ajija, Arivia Fikratuz Zakia, Rudi Purwono

**Affiliations:** Department of Economics, Faculty of Economics and Business, Universitas Airlangga, Indonesia

**Keywords:** ITPC, Non-oil and gas exports value, Indonesia, Global value, Developing country, Developed country

## Abstract

The opening of the Indonesian Trade Promotion Center is one of the policies implemented by the government to increase Indonesia's non-oil and gas exports. However, fluctuations in the value of non-oil exports make the role of the ITPC doubtful. This study aims to analyze the impact of Indonesia Trade Promotion Center as Export Promotion Agency (EPA) on the value of Indonesia's non-oil and gas exports globally, to developing countries, and developed countries from 2000 to 2018. The method used in this study is the Random Effect Model and gravity model. Estimation results show that EPA Indonesia (ITPC) has a positive and significant effect on the value of Indonesian non-oil exports in all models. Foreign direct investment (FDI) has a positive effect and significant for global and developing countries model. GDP per capita and Free Trade Agreements have a significant positive effect on non-oil exports in all models. The geographical distance variable has a negative impact for Indonesian non-oil exports in all models, except for the developed countries model.

## 1 Introduction

After World War II, the world economy experienced a postwar boom period that occurred from 1951 to 1970–1975 (Stephen and Juliet, 1992). According to Grieco (2001), One of the factors causing this economic expansion to occur is the high export value which is influenced by three things, namely the increase in intra-industrial trade, many developing countries have begun to participate in the world economy such as Southeast Asia and East Asia, and regional trade integration such as NAPHTHA.

Middleton (2000) states that the post-war boom ended in early 1973. The 1973 recession was marked by the collapse of the Bretton Wood System and the oil crisis that occurred as a result of the embargo imposed by the Organization of the Arab Oil Exporting Countries (OAPEC) against countries that were allies of Israel in the Yom Kippur War. During the 1970s, the price of oil continued to experience a significant increase until its peak occurred in 1980. The crisis and oil spillover that occurred from 1970 to 1980 had an impact on the trade policy of Indonesia as an oil-exporting country. Indonesia's export value is dominated by the oil and gas sector, so its performance depends on world oil production and prices. After the oil crisis and embargo ended, Rahmaddi dan Ichihashi (2011) and Soehoed (1988) stated that along with the fluctuating trend of oil prices, in 1969 the Indonesian government started the first industrialization phase focused on an import substitution strategy. The value of Indonesia's oil and gas exports continued to increase with the highest growth in 1974 at 88.51 percent, while non-oil and gas exports experienced negative growth of 19.55 percent. The value of non-oil and gas exports continues to decrease because of the imposition of import substitution. The positive growth trend for oil and gas continued to increase until its peak occurred in 1982 as a result of the decline in the value of oil production by OPEC to suppress price declines. However, this policy led to an increase in oil production by countries outside OPEC and many countries began to change the oil to other fuels.

Indonesia is faced with the problem of the dominance of high oil and gas exports. The magnitude of the dominance of oil and gas exports on Indonesia's total exports resulted in a decrease in the total export value of 3.29 percent as a result of the decline in the value of oil and gas exports by 10.11 percent. The use of the proceeds from oil exports to finance the fulfillment of imported raw materials and the number of funds needed for industrial development based on import substitution is considered a burden to the state. In 1982, the government then changed the ISI strategy to an export promotion strategy and export-based economic growth.

The combination of the export promotion strategy and export-led growth is expected to boost economic growth by increasing the role of exports in the economy, especially from the demand side. In this strategy, the industry changes the production orientation from the substitution of imported goods to export-oriented production. The increase in the value of non-oil and gas exports is an alternative so that the growth in the total value of exports continues to be positive. As shown in Table 1 (Appendix), the world oil price was at its lowest price in 1986, which resulted in a decline of 15.45 percent of Indonesia's oil and gas exports. On the other side, Indonesia's non-oil and gas exports increased by 39.82 percent and the total value of exports increased by 2.83 percent. Then in 1987, oil and gas exports grew by 7.53 percent, while non-oil and gas exports increased by 35 percent.

One of the policies contained in the export promotion strategy is the establishment of an Export Promotion Agencies (EPA). Hayakawa et al. (2014)stated that the establishment of representative offices or EPAs abroad is a way to increase export-based economic growth (export-led growth). The opening of representative offices abroad for the first time in Finland was in 1919 and became a policy followed by many other countries in improving export performance and reducing the problem of trade balance deficits. In a survey conducted by Lederman et al. (2006), there are at least 88 export representative offices in 116 countries, and it continues to increase until now.

The export promotion center established by the Indonesian government is called the Indonesian Trade Promotion Center (ITPC) which acts as a representative for the National Export Development Agency of the Ministry of Trade which has offices in several accredited countries. ITPC was founded on July 29, 1982, with the cooperation of the Ministry of Trade, Ministry of Industry, and the Ministry of Foreign Affairs. ITPC was temporarily closed due to the 1998 monetary crisis and reopened in 2000 in nine countries. Along with the development of diplomacy and export performance, ITPC was opened in eighteen countries, namely in Barcelona, Spain at the end of 2008; Budapest, Hungary in 2005; Busan, South Korea at the end of 2008; Chennai, India at the end of 2008; Chicago, United States at the end of 2008; Dubai, the United Arab Emirates in 2005; Hamburg, Germany in 2006; Jeddah, Saudi Arabia, in mid-2009; Johannesburg, South Africa in 2005; Lagos, Nigeria at the end of 2008; Los Angeles in 2005; Lyon, France at the end of 2008; Mexico City, Mexico at the end of 2008; Milan, Italy in mid-2007; Osaka, Japan in 2004; Santiago, Chile at the end of 2008; Sao Paulo, Brazil, in 2005; Sydney, Australia in 2005; and Vancouver, Canada in late 2008.

In Export Newsletter Ministry of Trade 2009, the opening of ITPCs in several countries is aimed at intensifying market breakthroughs, opening up a network of trade relations, facilitating the business world in export promotion activities, and marketing abroad. The office placement is based on government policies to diversify into non-traditional markets in line with the increase in existing traditional markets. There are various export promotion activities carried out by ITPC, such as exhibitions, business matching, and commercial diplomacy. The ITPC is intended to assist domestic business actors in accessing foreign markets as well as assisting importers from accredited countries to obtain information on Indonesian products.

Many countries have benefited from opening export promotion offices overseas. Empirical research has been conducted to determine the impact of export promotion strategies, especially EPA strategies. EPA is stated to have a positive impact on export performance and the internationalization process of domestic companies, including research by Lederman et al. (2006) in 103 developed and developing countries; Martincus dan Carballo (2008) in Peru; Rose (2007) in 22 countries; and Kang (2011) in South Korea.

EPA's contribution to Indonesia can be identified through the development of non-oil and gas export values. Since the emergence of the discourse on the reopening of the EPA, the value of Indonesia's non-oil and gas exports from 2001 to 2008 has continued to increase. Then, the government increased the number of Indonesian EPAs in 10 countries at the end of 2008. However, in 2009 the value of non-oil and gas exports decreased because of the global crisis. Non-oil and gas exports showed a fluctuating trend from 2010 to 2014.

The development of Indonesia's non-oil and gas export value in 18 main non-ITPC export destination countries is similar to the development of the non-oil and gas export value of ITPC accredited countries. This similarity includes the decline in the value of non-oil and gas exports in 2001 and 2009. The difference in the trend in the value of non-oil and gas exports occurred in 2012 in the form of a decline in the value of non-oil and gas exports to non-ITPC countries that occurred continuously until 2015, while non-oil exports -Oil and gas to ITPC accredited countries had experienced an increase in 2014. This fluctuation in the value of non-oil and gas exports occurred due to a decrease in global demand and a decrease in commodity prices and the competitiveness of Indonesian exports.

**Figure 1:**
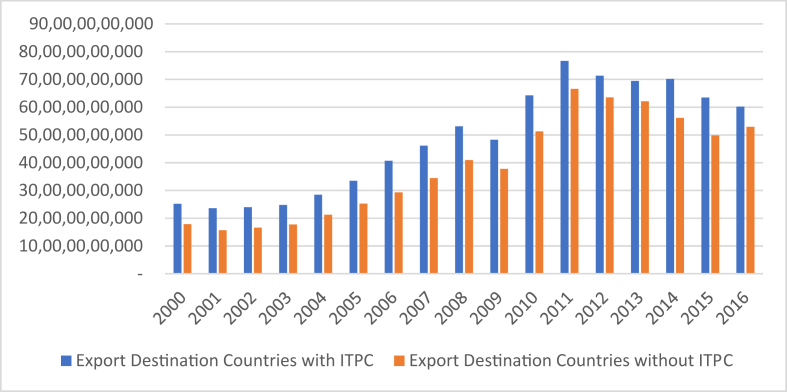
Development of Indonesia's Non-Oil and Gas Export Value (US $) to the 36 Main Export Destination Countries, 2000–2016.

Source: UN Comtrade (2019), compiled.

In recent years, the effectiveness and role of ITPC as Indonesia's export promotion agencies in increasing exports to accredited countries has begun to be questioned. Figure 1 (Appendix) explain growth of Indonesia’ non-oil and gas export for both countries with ITPC (accredited countries) and non-ITPC countries. From the graph, it can be concluded that from 2012 until 2016 the value of Indonesia's non-oil and gas exports to accredited countries continued to decline, and only in 2014 showed an increase. Similar conditions occurred in the eighteen main export destination countries that did not have ITPC offices (non-ITPC countries). From 2012 to 2016, Indonesia's non-oil and gas exports to non-ITPC countries experienced a decline (2012–2015) but then rise in 2016.

The deteriorating export performance has caught the attention of the Indonesian government. In the Ministry of Trade Meeting in January 2018, President Joko Widodo stated that he would close down the ITPC which did not make a large contribution to Indonesia's export growth because it absorbed a large enough budget (Hermansyah, 2018). Research on the influence of ITPC on exports conducted by Hapsari dan Haqqi (2017) stated that the ITPC has succeeded in bridging Indonesian sellers and buyers from the United States and in increasing Indonesia's non-oil and gas exports to America.

Research by Sinaga (2016) found that ITPC as Indonesia's EPA has a positive effect on the total export value, but does not have a significant impact on the export value of footwear. Subsequent research was carried out. Ulfah (2018) explained that the establishment of the Indonesian EPA had a significant impact on the total export value and exports of processed food. Other empirical research finds that EPA in some countries does not have a big influence on export growth. Martin (2003) stated that there is no relationship between exports and the presence of the American EPA in Japan. Cadot et al. (2015) in Tunisia in his research found that the effect of EPA (FAMEX) on exports is temporary for middle-class businesses and has a positive impact on small and large-scale businesses only.

Based on these descriptions, it is important to study the effect of ITPC as Indonesia's EPA on export value. Thus, the research aims to analyze the impact of the opening of the Indonesian Trade Promotion Center on the value of Indonesia's non-oil and gas exports. Besides, this study analyzes the impact of the opening of the ITPC on the value of Indonesia's non-oil and gas exports to developing countries and developed countries.

## 2 Literature review

### 2.1 International trade

Modern international trade theory began when economists Heckscher (1919) and Bertil Ohlin (1933) presented an explanation of international trade that could not be explained in the theory of comparative advantage. The classical theory of comparative advantage explains that international trade can occur because of differences in labor productivity between countries (Salvatore, 2001). However, this theory does not explain the causes of differences in productivity.

According to the HO theory, there is a difference in productivity due to the number or proportion of production factors owned (endowment factors) by each country, causing differences in the price of goods produced. The HO theory predicts that countries will export goods whose production uses the abundant factor endowments they have, as well as import goods whose production requires factor endowments that are not available in abundance in their countries. Vernon (1966) developed The Product Cycle Theory as his critic towards to the Heckscher-Ohlin's theorem. His point of view for his theory based on US perspective (at this point, developed countries).

The PCT is divided into three stages: new-product stage, maturing-product stage, and standardized-product stage (Appleyard et al., 2011). At first, the product is produced in developed countries and consumed in the developed country. After that, standardization and mass production begin and then exported to other developed countries of the maturing-product stage. The third stage is standardized-product stage where the trade pattern is shifted: developed countries become importer of the product, and developing countries become their exporter country.

Lam (2015) mentioned that as developed countries entered the first stage, developing countries imported the product. As domestic demand of the developing countries rises, the domestic production of the product started. In the next stage, developing countries substitutes the imported product to their domestic product as stated in Balassa's five-stages of development. But then, the domestic demand's growth of the product tends to decrease, and the developing countries begin to export the product. The third stage of product in developing countries is in line with the third stage of Vernon's product cycle theory of developed countries. Related to this stage, developing countries must strengthen their competitiveness and higher their export to fulfill the demand from developed countries. Therefore, developing country uses various policies to increase their exports, especially to developed countries. These policies include participation in FTAs and the opening of export promotion agents in export destination countries.

#### 2.1.1 Foreign direct investment

According to Samuelson dan Nordhaus (2010, p. 198), the investment includes the addition of capital stock or goods in a country, such as buildings, production equipment, and inventory items within one year. A form of foreign direct investment (foreign direct investment) is a foreign company that has a controlling interest in a local company, buys or sets up new factories abroad, moves funds abroad for company expansion, or re-invested revenue from a foreign subsidiary (Carbaugh, 2004, p. 311, p. 311). Krugman et al. (2012, p. 183) divides FDI into vertical FDI and horizontal FDI. Vertical FDI tends to increase the value of a country's exports more than horizontal FDI. However, horizontal FDI is not always focused on the domestic market only but is also oriented towards foreign markets (doing exports).

FDI plays an important role in the development process, especially in developing countries (Widyananda and Sari, 2020). In its effect on exports, FDI plays a role in several ways, namely direct and indirect or the spillover effect. The direct role of FDI in increasing exports can be seen through the export value of MNC itself. Markusen (1998) stated that if the investment motive is trade-oriented, investors will invest foreign direct investment to find low production costs so that there will be an increase in production and an increase in the value of trade (exports). This is also related to the impact of FDI as export-driven, namely with the presence of FDI, especially export-oriented FDI, the government tends to reduce export barriers so that it has an impact on the growth in export value which is quite high to increase economic growth (Rahmaddi and Ichihashi, 2013). *The spillover effect* of FDI to exports is an increase in domestic market competition which in turn encourages domestic companies to increase efficiency and productivity (Zhang and Song, 2001). Second, there is knowledge spillover in the form of increasing exports by domestic companies through learning from export activities carried out by MNCs (Haddad and Harrison, 1993).

### 2.2 Gravity model

The gravity model is one of the popular models used in international trade analysis. The gravity model was adapted from Newton's law of gravity by Tinbergen in 1962 (Anukonwattaka, 2016). The gravity model plays a role in understanding the value of trade between countries as well as showing the barriers to free trade (Krugman et al., 2012, p. 12). The difference between Newton's gravity model and Tinbergen's gravity model is that the mass variable is replaced by GDP, while the distance variable is replaced by trade costs (such as distance between national capitals, discussion, bilateral tariff barriers, etc.). The gravity model equation can be written as follows:

(1) $$X_{ij}=C\frac{Y_{i}Y_{j}}{t_{ij}}$$

Information:

$X_{ij}$: export or trade from the country I to country j.

*C*: constant.

$Y_{i}$: GDP of the country *i*.

$Y_{j}$: GDP of the country *j*.

$t_{ij}$: *trade costs* between the country I and country j.

The gravity model assumes that the greater the value of the national income of the exporting country, the greater the ability to produce and export goods. Furthermore, the higher national income variable of the importing country indicates a greater need for goods and services, including the need to import goods from other countries. The distance variable becomes a proxy for transportation costs between countries, so it is assumed that the greater the distance between two countries, the lower the export value of country I to the export destination country j.

The gravity model has been used in various studies. Applications of the gravity model include research on the elasticity of trade volume, the impact of economic integration on trade value, and the impact of institutions and facilities on export value. In addition to the GDP variables for both countries and distance, some researchers added population variables to measure market size, free trade agreements, language, colonial links, landlocked, and boundaries between countries.

### 2.3 Economic integration

Economic integration is defined as reducing or even eliminating economic barriers between two or more countries (Pelkmans, 2006). Carbaugh (2004) defines economic integration as the stage of reducing barriers to trade, payment, and factor mobility. Economic integration results in the unification of two or more economies that are bound by regional trade agreements. Economic integration consists of a free trade area, customs union, the common market, and at the final stage is a monetary union such as the European Union using one currency for all member countries.

The establishment of a trade area is one of the discriminatory commercial policies to reduce or remove special trade barriers for member countries. The agreement regarding the reduction of tariff and non-tariff barriers only applies to countries that are members of economic integration (Salvatore, 2001).

#### 2.3.1 Export promotion agencies

Opening the first overseas export representative office in Finland in 1919 had a major impact on improving export performance and reducing the trade balance deficit problem. The success of the EPA has encouraged various countries to open export promotion agents (EPA). EPA, assisted by diplomatic representatives, is the main source of information in dealing with the problem of market failures and increasing trading activity (Gil-Pareja et al., 2008). Before exporting their products, prospective exporters should understand the preferences and conditions of consumers in the export destination country. However, the costs of obtaining information and marketing costs in foreign markets are quite large, especially for prospective local SME exporters. In this case, EPA as the country's representative office abroad helps prospective exporters understand and find new markets for their products. Facilities provided by the EPA (Lederman et al., 2010) consists of country image building. export support services, marketing, and market search and market survey.

The services that the EPA provides create externalities. EPA not only facilitates domestic exporters but provides information and assistance for overseas people who wish to import or invest in the EPA's home country. Positive externalities occur when the social benefits obtained are greater than the social costs and there is an increase in export performance, both in terms of the number of exporters, sectors, and the length of time to export goods to an accredited country (Lederman et al., 2010). The social cost of EPA can increase due the rent-seeking and misuse of information by unauthorized parties. Importers' databases, existing distribution chains, and services previously obtained by potential exporters from the EPA can be used by competitors (Hausmann and Rodrik, 2003).

Previous research stated that EPA affects product differentiation. Martincus dan Carballo (2008) stated that the opening of the EPA resulted in the emergence of extensive margins and intensive margins. The intensive margin is the growth in exports of old products to old export destination countries, while the extensive margin is an increase in exports of new products to old countries, growth in exports of old goods to new countries, and an increase in exports of new products to new countries. Crespi dan Alvarez (2000) concluded that the Chilean EPA has a positive influence on product diversification and market expansion.

Rose (2007) researches the role of embassies and consulates in increasing export value. The results of the study show that the opening of an embassy has a greater impact on exports than the addition of a consulate general. Lederman et al. (2010) researching the impact of EPA on the value of exports resulted in the conclusion that the per-capita EPA budget has a positive coefficient and is statistically significant in affecting the value of exports. Other research on the regional influence of the Spanish EPA on exports shows that the EPA has a positive impact on increasing the value of exports (Gil-Pareja et al. (2008) in Spain and Martincus dan Carballo (2008) in Peru). Kang (2011) concluded that the opening of KOTRA as the EPA of South Korea was an important factor and had a significant impact on the success of South Korean exports.

Sinaga (2016) in her research stated that the ITPC had a positive and significant impact on the total value of Indonesian exports, but not significant on exports of footwear commodities. Ulfah (2018) concluded that ITPC had a positive and significant impact on Indonesia's total exports and exports of processed food. Rose (2007) and Gil-Pareja et al. (2008) concluded that the distance variable has a negative and significant effect on the export value. Mahmood dan Jongwanich (2018) in Pakistan as well Kang (2011) in South Korea, it is argued that the geographical distance variable in this study has a negative and significant impact on export value.

The free trade variable or FTA has a positive and significant impact on export value (Gil-Pareja et al., 2008; Chiappini, 2016). Mahmood dan Jongwanich (2018) in his research stated that the in-effect Pakistan-China FTA is a free trade agreement that has the greatest impact on increasing the value of Pakistan's exports. The independent variable per-capita GDP also has a positive and significant impact on the increase in export value (Rose, 2007; Lederman et al., 2010).

FDI is stated to have a positive impact on the value of exports (Sun and Parikh, 2001; Rahmaddi and Ichihashi, 2013; Mijiyawa, 2015). Research by Kang (2011) in South Korea, Rahmaddi dan Ichihashi (2013) in Indonesia as well Mahmood dan Jongwanich (2018) in Pakistan shows that the exchange rate variable has a positive and significant impact on the value of exports. If the currency depreciates, there will be an increase in demand for goods from abroad, and the value of exports will increase.

Referring to the formulation of the problem, the theoretical basis, and previous research, the hypotheses for this study are as follows:

**H1.***Foreign direct investment* (FDI) has a positive effect on the value of Indonesia's non-oil and gas exports.

**H2.** The Gross Domestic Product per capita (GDP per capita) of the export destination countries (importing countries) has a positive effect on the value of Indonesia's non-oil and gas exports.

**H3.** The nominal exchange rate has a positive effect on the value of Indonesia's non-oil and gas exports.

**H4.** Geographical distance has a negative effect on the value of Indonesia's non-oil and gas exports.

**H5.** Free Trade Agreements (FTA) have a positive effect on the value of Indonesia's non-oil and gas exports.

**H6**. The Indonesian Trade Promotion Center (ITPC) has a positive effect on the value of Indonesia's non-oil and gas exports.

## 3 Data and research methods

This study aims to determine the impact of the ITPC on the value of Indonesia's non-oil and gas exports. The data used in this study are panel data for the period 2000 to 2018 eighteen ITPC accredited countries as well as eighteen main non-ITPC export destination countries based on data on the value of exports to the main destination countries of the Central Statistics Agency in 2019. The list of observed countries in the models of developing countries and developed countries is shown in Table 1 and the variable definitons stated in Table 2.

**Table 1:** List of observation states.

Developing Countries		Developed Countries	
ITPC accreditation	Non-ITPC	ITPC accreditation	Non-ITPC
South Africa	Philippines	United States of America	Netherlands
Saudi Arabia	Hong Kong	Australia	Belgium
Brazil	Cambodia	Hungary	Denmark
Chile	Malaysia	Italy	Finland
India	Myanmar	Japan	English
South Korea	Singapore	German	Poland
Mexico	Thailand	Canada	New Zealand
Nigeria	China	France	Sweden
United Arab Emirates	Vietnamese	Spanish	Greece

Source: Countries Economic Grouping, UNCTAD (2019).

**Table 2:** Variable definitions.

Variable Name	Definition	Source
Non-Oil and Gas Exports	Total exports minus oil and gas commodities (SITC 333, SITC 334, 335, SITC 34).	UN COMTRADE
*Foreign Direct Investment*	FDI from the export destination country to Indonesia	Ministry of Investment (before: Investment Coordi-nating Board or BKPM)
GDP per capita	The market value of all final goods and services produced in an export destination country divided by population of the destination country	World Bank
Nominal Exchange Rate	Rupiah nominal exchange rate against US $.	UNCTAD
Geographical Distance	Distance between national capitals	CEPII
*Free Trade Agreements*	Free trade agreement between Indonesia and export destination countries (signed and in effect)	ADB
*Indonesian Trade* *Promotion Center* (ITPC)	Center for the promotion of Indonesian exports abroad or Indonesia's export promotion agencies in certain export destination countries.	Ministry of Trade Indonesia

In the Trade Statistics of the Ministry of Trade (2017), non-oil and gas export commodities are all Indonesian export commodities minus oil and gas commodities which consist of crude oil (SITC 333), oil products (SITC 334 and 335), and gas (SITC 34). The non-oil and gas export variable used in this study is the value of non-oil and gas exports, due to the lack of data available on the volume of exports per commodity to each destination country.

The variable value of non-oil and gas exports, foreign direct investment, and GDP per-capita are expressed in US$ units. The data of those variables including nominal exchange rate and geographical distance are converted into a natural logarithm to homogenize the data. Overall, the descriptive statistics of the variables used in this study are summarized in Table 3.

**Table 3:** Description of variable statistics.

Variable	n	Mean	Min	Max	Std. Deviation
**Global Value**					

Non-oil and gas exports	684	20.66348	17.24305	23.90979	1.508182
*Foreign Direct Investment*	684	12.56422	0	22.94173	7.899315
GDP per capita	684	9.46837	4.92120	11.12947	1.413935
Nominal exchange rate	684	9.23483	9.03860	9.56359	0.1650989
Geographical Distance	684	8.84631	6.78687	9.73290	0.7466588
**Developing Countries**					

Non-oil and gas exports	342	20.90931	17.6367	23.90979	1.431267
*Foreign Direct Investment*	342	10.68903	0	22.94173	8.731371
GDP per capita	342	8.72816	5.83522	11.05487	1.335794
Nominal exchange rate	342	9.23483	9.03860	9.56359	0.1652199
Geographical Distance	342	8.44666	6.78687	9.73290	0.8507139
**Developed Countries**					

Non-oil and gas exports	342	20.41764	17.24305	23.63181	1.544716
*Foreign Direct Investment*	342	14.43941	0	22.40983	6.456871
GDP per capita	342	10.51739	9.05091	11.0498	0.4388394
Nominal exchange rate	342	9.23483	9.03860	9.56359	0.1652199
Geographical Distance	342	9.24597	8.59612	9.70327	0.2693118

Source: STATA 13 regression results.

Note: Data used for estimation and stated in descriptive statistics already transformed into natural logarithm.

The empirical analysis in this study is divided into three parts. The first analysis uses global value data consisting of 18 ITPC accredited countries and 18 non-ITPC main export destination countries. The second analysis uses data from developing countries consisting of nine developing countries accredited by ITPC and nine developing countries that are not ITPC-accredited. Meanwhile, the third analysis uses data from developed countries consisting of nine ITPC accredited developed countries and nine non-ITPC developed countries. The purpose of this data sharing is to determine the specific and general characteristics of each country category.

Table 4 shows the descriptive statistics of the dummy free trade area and ITPC variables. Based on the table, for the global value model, the percentage of countries that have the same FTA membership as Indonesia is 36% or 13 countries, while the non-FTA countries are 64% or 23 countries. ITPC accreditation countries are 50% or 18 countries and non-ITPC countries are 50% or 18 countries. In the model of developing countries, there are 11 countries with a percentage of 73% that are members of the same FTA as Indonesia, while in the developed countries model there are two countries or 11% of the countries in the same FTA. Then, the models of developing countries and developed countries have the same number of countries with ITPC accreditation, namely 50% or 18 countries.

**Table 4:** Descriptive statistics of dummy variables.

Variable	Frequency 1		Frequency 0	
	Frequency	Percentage	Frequency	Percentage
**Global Value**				

Indonesia Trade Promotion Center	18	50	18	50
Free Trade Area	13	36	23	64
**Developing Countries**				

Free Trade Area	11	73	7	27
Indonesia Trade Promotion Center	9	50	9	50
**Developed Countries**				

Free Trade Area	2	11	16	89
Indonesia Trade Promotion Center	9	50	9	50

Source: STATA 13 regression results.

Because the data used in this study is panel data, the empirical analysis used is a panel regression model. According to Gujarati and Porter (2009: 592), data panel was judged to be able to provide heterogeneity, given the characteristics of the variables or subject-specific variables such as country, individuals, companies, and others. Panel data is also considered more informative, more varied reduces collinearity problems between independent variables, is more efficient, and has more degrees of freedom. Thus, panel data is considered to be better at detecting and measuring effects that cannot be measured by time-series data alone or by cross-section data alone so it is suitable for examining the dynamics of change because it can minimize the occurrence of bias. In general, the empirical analysis in this study is as follows:

(2) $$\ln\;NFEX_{ijt}=\beta_{0}+\beta_{1}lnFDI_{jit}+\beta_{2}lnPCP_{jt}+\beta_{3}lnER_{it}+\beta_{4}lnDIST_{ijt}++\beta_{5}FTA_{ijt}+\beta_{6}ITPC_{ijt}+\varepsilon_{it}$$

Information:

$NFEX_{ijt}$ : the value of Indonesia's non-oil and gas exports i to country j year t.

$\beta_{0}$ : constant.

$FDI_{jit}$ : *foreign direct investment* country j to Indonesia i year t.

$PCP_{jt}$ : GDP per capita country j year t.

$ER_{it}$ : the nominal exchange rate of Indonesia against the US dollar, year t.

$DIST_{ijt}$ : the geographical distance between Indonesia country i against country j year t.

$FTA_{ijt}$ : dummy variable The FTA between Indonesia i and country j year t, is worth 1 if the two countries are in the same Free Trade Agreements.

$ITPC_{ijt}$ : EPA office dummy variable country i in country j in year t, is worth 1 if Indonesia has an EPA representative in the export destination country.

$\varepsilon_{it}$ : error terms.

## 4 Results and discussion

The panel data selected estimation model is determined through three tests, namely the Chow test to test whether PLS or FEM is chosen to be the best model, the Hausman test to determine between FEM or REM as the best model, and the Lagrange Multiplier test to determine between REM or PLS. The results of the best model selection test are described in Table 5.

**Table 5:** Random effect model estimation results.

Variable Independent	Dependent Variable: lnNFEX					
	Global Value		Developing Countries		Developed Countries	
	(1)	(2)	(1)	(2)	(1)	(2)
Constant	22,092 ∗∗∗ (1,6911)	10,432 ∗∗∗ (0.7303)	14,563 ∗∗∗ (2,2857)	7,453 ∗∗∗ (1,5591)	2,3265 (6,338)	1,6109 (1.9943)
lnFDI	0.0066 ∗∗ (0.0027)	0.0076 ∗∗∗ (0.0028)	0.0148 ∗∗∗ (0.0054)	0.0179 ∗∗∗ (0.0056)	0.0057 (0.0040)	0.0059 (0.0040)
lnPCP	0.9633 ∗∗∗ (0.0327)	0.9191 ∗∗∗ (0, 0335)	1.1187 ∗∗∗ (0.0868)	1.0539 ∗∗∗ (0.0876)	1,8115 ∗∗∗ (0.1972)	1.8026 ∗∗∗ (0.1965)
lnER	0.1203 (0.0783)	0.1402 ∗ (0.0818)	0.3792 ∗∗ (0.1807)	0.3993 ∗∗ (0.1858)	-0.0401 (0.1038)	0.0404 (0.1042)
lnDIST	-1,3407 ∗∗∗ (0.1768)		-0.8761 ∗∗∗ (0.2099)		-0.0876 (0.6537)	
FTA (dummy)	0.1773 ∗∗∗ (0.0597)	0.2696 ∗∗∗ (0.0610)	0.3324 ∗∗∗ (0.1132)	0.4833 ∗∗∗ (0.1115)	0.3954 ∗∗∗ (0.0915)	0.3956 ∗∗∗ (0.0918)
ITPC (dummy)	0.2189 ∗∗∗ (0.0369)	0.2066 ∗∗∗ (0.03855)	0.5047 ∗∗∗ (0.0793)	0.4298 ∗ (0.0799)	0.3809 ∗∗∗ (0.0478)	0.3822 ∗∗∗ (0.0480)
Adj R	0.77	0.76	0.68	0.67	0.51	0.51
F	0.0000	0.0000	0.0000	0.0000	0.0000	0.0000
Observation (n)	684	684	342	342	342	342

Source: STATA 13 data regression results.

Note: Column (1) shows the regression results with the geographic distance variable, while column (2) shows the regression results without the geographic distance variable. Numbers in brackets () indicate the standard error value; ∗∗∗, ∗∗, and ∗ show statistically significant variables at α 1%, 5%, and 10%, respectively.

The FEM regression model of global value and developed countries in STATA cannot be used because the geographical distance variable cannot be estimated (omitted variable). Therefore, the Lagrange Multiplier test was performed and concluded that the best alternative was the Random Effect Model.

REM estimation results for the analysis of global value, developing countries, and developed countries in Table 5 show a probability value of F-statistic of 0.000. This probability value is significant at the 5% significance level, so it can be concluded that the variables of foreign direct investment, GDP per capita of the importing country, nominal exchange rates, geographical distance, free trade agreements, and ITPC as Indonesia's EPA simultaneously affect the value of non-oil and gas exports. Indonesia.

Specifically, in the analysis of global value data, it is known that Foreign Direct Investment, GDP per capita of importing countries, FTA, and ITPC have a positive and significant effect on Indonesia's non-oil and gas exports. The nominal exchange rate variable has a positive but insignificant effect on Indonesia's non-oil and gas exports. The geographical distance variable has a negative and significant impact on the value of non-oil and gas exports. On data analysis of developing countries, Foreign Direct Investment, GDP per capita, nominal exchange rate, FTA, and ITPC has a significant positive effect on the value of Indonesia's non-oil and gas exports. Distance-geographical has a negative and significant effect on the value of Indonesia's non-oil and gas exports. Then, the estimation of developed countries shows, Foreign Direct Investment, and the nominal exchange rate have a positive but insignificant impact on the value of Indonesia's non-oil and gas exports. GDP per capita, FTA, and ITPC have a positive and significant effect on the value of Indonesia's non-oil and gas exports. Geographical distance has a negative but insignificant effect on the value of Indonesia's non-oil and gas exports.

The estimation results in this study indicate that FDI has a positive effect on Indonesia's non-oil and gas exports in the three models. The estimation results of the global value model show that FDI has a coefficient of 0.0066, which means that an increase of 1% in the value of FDI in the country of export to Indonesia will increase the value of Indonesia's non-oil and gas exports by 0.007% assuming other variables are constant. In the model of developing countries, FDI has a coefficient of 0.0148, which means an increase of 1% in the value of FDI in developing countries to Indonesia will increase the value of non-oil and gas exports by 0.015%, assuming other variables are constant. The FDI coefficient in the developed countries model is 0.0057, which means that a 1% increase in FDI from developed countries to Indonesia increases the value of non-oil and gas exports by 0.006%, other variables are constant.

The effect of FDI on Indonesia's non-oil and gas exports in the global value model and developing countries are in line with previous studies (Sun and Parikh, 2001; Rahmaddi and Ichihashi, 2013; Mijiyawa, 2015; Chiappini, 2016; Duong et al., 2019). FDI in both models has a significant effect on Indonesia's non-oil and gas exports, however, the FDI coefficient is relatively small compared to the coefficient on other independent variables. This implies that FDI in the non-oil and gas sector in Indonesia tends to be motivated by domestic market penetration or tariff-jumping type investment compared to investment for export orientation. Also, the coefficient value that is not too large indicates that Indonesia has not had a significant impact on export oriented FDI. This is confirmed by the research Negara dan Adam (2012) that Indonesia has not maximized the positive impact of FDI.

In the global value model, GDP per capita has a coefficient of 0.963, which means that with every 1% increase in GDP per capita of export destination countries, the value of Indonesian exports has increased by 0.96%, other variables are constant. The estimation results in the model of developing countries produce a per-capita GDP coefficient of 1.117, which means an increase of 1% of GDP per capita increases the value of Indonesia's non-oil and gas exports by 1.12%, other variables are constant. For models of developed countries, GDP per capita has a coefficient of 1.811, which means that an increase of 1% of GDP per capita in developed countries means that Indonesia's non-oil and gas exports increase by 1.81%, other variables are constant. The estimation results of the GDP per capita of the export destination countries on the value of non-oil and gas exports in this study indicate that the GDP per capita has a positive and significant effect on all models. The results support previous research which states that GDP per capita has a positive and significant effect on export value (Lederman et al., 2010; Sinaga, 2016; Ulfah, 2018).

The estimation of the global value model produces a nominal exchange rate coefficient of 0.120, so it can be concluded that the nominal exchange rate depreciation increases the value of non-oil and gas exports by 0.12%. Regression in the model of developing countries produces a coefficient of 0.379 so that it can be concluded that when there is a depreciation of the exchange rate of 1%, it will increase the value of Indonesia's non-oil and gas exports to developed countries by 0.38%. The developed countries model's coefficient for exchange rate is -0.041, which means that when the exchange rate depreciates, Indonesia's non-oil and gas exports to developed countries decline by 0.04%.

Thus, it can be concluded that the nominal exchange rate variable has a positive effect on the value of Indonesia's non-oil and gas exports in the global value model and developing countries. Appleyard et al. (2011) stated a similar thing that in the long run, the depreciation will cause an increase in exports. The estimation results of this study for the nominal exchange rate in both models support previous research which concluded that the exchange rate has a positive impact on export value (Sun and Parikh, 2001; Kang, 2011; Rahmaddi and Ichihashi, 2013; Mijiyawa, 2015; Duong et al., 2019).

Distance between the capital city of two countries (capital of Indonesia to export destination country) is used as a proxy for transportation costs. Regression in the global value model results in a geographic distance coefficient of -1.341 which means that adding 1% to the distance (in kilometer), the cost of transportation for Indonesia to export destination countries increases by 1% (as mentioned above, geographic distance between two capital as a proxy of transportation cost), the value of Indonesia's non-oil and gas exports decreases by 1.34%, other variables are constant. The estimation results in models in developing and developed countries show a coefficient of -0.876 and -0.088. This means that a 1% increase in distance as a proxy of transportation costs will reduce Indonesia's non-oil and gas exports to developing countries by 0.88% and to developed countries by 0.08%, other variables are constant. We can concluded that the greater the distance, the greater the transportation costs, the lower trade happen between two countries.

Geographical distance in the global and developing models has a negative coefficient and same conclusion with previous research (Rose, 2007; Gil-Pareja et al., 2008; Kang, 2011; Mahmood and Jongwanich, 2018). Distance does not have a significant impact on the models of developed countries according to the study Bhagwati (2013) which states that technological advances can eliminate the cost of transportation between countries. The geographic distance coefficient in the model of developed countries has a smaller value when compared to the global value model and developing countries. This is because, during the study period Indonesia's non-oil and gas exports were dominated by United States and Japan which is included as developed countries category.

The dummy variable FTA in the global value model has a coefficient of 0.177, which means that Indonesia's non-oil and gas exports to importing countries that are members of the same signed and in effect FTA are 18% greater than non-FTA importing countries, other variables are constant. The estimation results in models in developing and developed countries show the FTA coefficient values of 0.33 and 0.39. Indonesia's non-oil and gas exports to developing countries of export destinations joined in the same FTA were 33% greater than exports of non-FTA joined countries, other variables are constant. Meanwhile, Indonesia's non-oil and gas exports to developed countries under the same FTA were 39% greater than developed non-FTA countries.

Based on the estimation results, the impact of FTAs on Indonesia's non-oil and gas exports to developed countries is greater than in developing countries. One of the reasons is because Japan is the main destination country for Indonesia's non-oil and gas exports and has the same FTA membership as Indonesia. Overall, the FTA dummy variable has a positive and significant impact on the value of Indonesia's non-oil and gas exports, in line with previous research that FTA has a positive impact on export value (Sinaga, 2016; Mahmood and Jongwanich, 2018; Ulfah, 2018).

The ITPC dummy variable in the estimation of the global value model has a coefficient of 0.22, which means that if there is ITPC in the export destination country, the value of Indonesia's non-oil and gas exports is 22% higher than a country without ITPC, assuming ceteris paribus. The estimation in the model of developing countries results coefficient of 0.50, which means that if there is an ITPC in a developing country, the value of Indonesia's non-oil and gas exports is 50% higher than non-oil and gas export to developing countries without ITPC, assuming ceteris paribus. The ITPC coefficient in the developed countries model is 0.38, which means that if the developed country (Indonesia's trade partner) has ITPC, the value of Indonesia's non-oil and gas exports is 38% greater than developed country without ITPC in it, assumption of ceteris paribus.

In the 2015–2019 RPJMN, one of the government's efforts to increase the value of non-oil and gas exports is to increase the effectiveness of market intelligence, promotion, and export assistance, all of which are part of the main tasks of ITPC. The opening of the ITPC, which has a positive and significant impact on the value of Indonesia's non-oil and gas exports, is in line with the 2015–2019 RPJMN. Research on the influence of ITPC as Indonesia's EPA has been conducted by Sinaga (2016) and Ulfah (2018) concluded that there was a positive and significant effect of the ITPC in increasing export value. Furthermore, the results of this study support the findings Hapsari dan Haqqi (2017) which stated that the ITPC Chicago was proven to have a positive effect on the increase in the trade balance of non-oil and gas commodities from Indonesia to America. Previous research stated that EPA has a positive and significant impact on exports (Gil-Pareja et al., 2008; Martincus and Carballo, 2008; Lederman et al., 2010; Kang, 2011).

The analysis shows that the ITPC has a significant effect on the value of Indonesia's non-oil and gas exports. The positive impact of ITPC on significant non-oil and gas exports can be a consideration for the government to continue the ITPC, both for developing countries and developed countries. Regarding the value of the ITPC coefficient, the effect of ITPC presence in developing countries is bigger than ITPC in developed countries. So, to increase Indonesia's non-oil and gas export, the government can build or establish new ITPC in developing countries. For the developed countries, if the government consider open more ITPCs in developed countries, it can higher the non-oil and gas export even though the coefficient of ITPC in developed countries model is smaller than developing countries model. Furthermore, the opening of the ITPC in developed countries was strengthened by the results of the estimation that geographical distance did not have a significant impact on non-oil and gas exports, as mostly the geographic distance from Indonesia to developed countries is further than Indonesia to developing countries.

In the model of developing countries, the variable that has a large impact on non-oil and gas exports is GDP per capita. Per-capita GDP tends to increase so that the need for non-oil and gas imports by developing countries is increasing. The addition and improvement of ITPC performance are needed to facilitate and encourage developing countries to import Indonesian non-oil and gas products. Policies such as export promotion and participation in trade fairs should be increased. Then, additional staff is needed, especially home staff to increase the effectiveness of ITPC. Cooperation between the ITPC and the Trade Attache needs to be increased so that the target of increasing the value and share of the export market can increase.

## 5 Conclusion

This study aims to analyze the impact of the opening of the ITPC on the value of Indonesia's non-oil and gas exports. Variable of interest in this research is ITPC, and the other explanatory variables chosen for this study are foreign direct investment, GDP per-capita, nominal exchange rate, geographical distance, and free trade agreements (FTA). FDI affect export value of Indonesia both in indirect and direct ways. GDP per-capita indicates purchasing power of importing countries (trade partners). Nominal exchange rate affect exports, the depreciation of exchange rate will encourage foreign costumers/countries to increase their import towards Indonesia. Farther the distance of two countries will increase the transportation cost and lower the exports. To reduce trade barriers, countries have some free trade agreements, so their trade (especially their export) wills goes up.

The results of the study show that the ITPC has a positive and significant impact on Indonesia's non-oil and gas exports in the global value model, developing countries, and developed countries. In global value model, the existence of ITPC can increase the value of Indonesia's non-oil and gas exports by 22%. In the developing country model, it is able to increase Indonesia's exports by 50%, and in the developed country model, the presence of the ITPC will increase Indonesia's exports by 38 percent. Based on the increase in export value, it is recommended that the Indonesian government should maintain and expand the presence of ITPC to the developing countries and keep maintaining ITPC in developed countries and global markets.

Specifically, FDI has a positive impact on Indonesia's non-oil and gas exports. However, the value of the FDI coefficient is small when compared to other variables. It is suspected that FDI entering Indonesia is more oriented towards domestic demand than exports and is a tariff-jumping investment in nature. The variable GDP per capita of importing countries has a significant positive effect on the value of Indonesia's non-oil and gas exports in all analysis models. The increase in GDP per capita of importing countries increases the value of import demand, thereby increasing Indonesia's non-oil and gas exports. The nominal exchange rate variable has a significant positive effect on the value of Indonesia's non-oil and gas exports in the global value model and developing countries. The geographic distance variable hurts the value of Indonesia's non-oil and gas exports in all models. Geographical distance has no significant impact on non-oil and gas exports to developed countries. Meanwhile, variable FTA have a positive and significant effect on Indonesia's non-oil and gas exports in all models.

Overall, the results of this research can be a consideration for Indonesia's government to continue current ITPC. In addition, the coefficient of ITPC in developing countries is larger than developed countries. So, the government can continue and establish new ITPC(s) in developing countries. Even though the impact of ITPC in developed countries smaller than developing countries, the government may consider to expanding ITPC office in another potential countries. Mostly, developed countries located far away from Indonesia, but the geographical distance in this research shows that it does not have a significant impact (and the coefficient much smaller than the developing countries model). From the results this research has provided, Indonesia can reach a higher value as well as expand it is market for non-oil and gas exports. Therefore, the presence of ITPC in a country will increase Indonesia's market intelligence in that country and make it easier for Indonesia's exporters to export their product to accredited country (country which ITPC established). However, it is important for the government to not focuses on aggregate value of non-oil and gas export, but to be oriented to the value of each commodities related to export diversification. The data used for this research is an aggregated data and does not specify the commodities. Further research may analyze the impact of ITPC on non-oil exports in more detailed commodities (using data of 2-digit or 3-digit HS). According to other variable used such as FTA, future research might use a discrete variable of FTA based on each FTA signed and in effect (such as AFTA, ACFTA, etc).

## Declarations

### Author contribution statement

Shochrul Rohmatul Ajija: Conceived and designed the experiments; Performed the experiments; Contributed reagents, materials, analysis tools or data; Wrote the paper.

Arivia Fikratuz Zakia: Analyzed and interpreted the data; Contributed reagents, materials, analysis tools or data; Wrote the paper.

Rudi Purwono: Conceived and designed the experiments; performed the experiments.

### Funding statement

This work was supported by 10.13039/501100008463Universitas Airlangga, Riset Mandat 2020.

### Data availability statement

Data will be made available on request.

### Declaration of interests statement

The authors declare no conflict of interest.

### Additional information

No additional information is available for this paper.

## Figures and Tables

**Table:** Table 1: Indonesia's Export Value, 1972–1087 (million LCU)

Year	Oil and Gas		Non-oil and gas		Total	
	Value (million LCU)	Growth (%)	Value (million LCU)	Growth (%)	Value (million LCU)	Growth (%)
1972	1,257,407	55.00	1,190,593	(2.79)	2,448,000	20.24
1973	1,461,472	16.23	1,455,528	22.25	2,917,000	19.16
1974	2,755,034	88.51	1,170,966	(19.55)	3,926,000	34.59
1975	3,260,671	18.35	1,094,329	(6.54)	4,355,000	10.93
1976	3,900,302	19.62	1,648,698	50.66	5,549,000	27.42
1977	4,336,324	11.18	2,041,676	23.84	6,378,000	14.94
1978	4,575,736	5.52	2,095,264	2.62	6,671,000	4.59
1979	5,089,874	11.24	2,716,126	29.63	7,806,000	17.01
1980	6,169,672	21.21	2,416,328	(11.04)	8,586,000	9.99
1981	6,619,666	7.29	1,675,334	(30.67)	8,295,000	(3.39)
1982	7,712,331	16.51	1,645,669	(1.77)	9,358,000	12.81
1983	6,985,078	(9.43)	2,162,922	31.43	9,148,000	(2.24)
1984	6,771,856	(3.05)	2,679,144	23.87	9,451,000	3.31
1985	6,086,949	(10.11)	3,053,051	13.96	9,140,000	(3.29)
1986	5,146,778	(15.45)	4,252,222	39.28	9,399,000	2.83
1987	5,534,094	7.53	5,763,906	35.55	11,298,000	20.20

Source: World Development Indicator (2019).

Additional note: Indonesia's LCU (in rupiah).

( ) is a negative value.
